# FAD: Fine-Grained Adversarial Detection by Perturbation Intensity Classification

**DOI:** 10.3390/e25020335

**Published:** 2023-02-11

**Authors:** Jin-Tao Yang, Hao Jiang, Hao Li, Dong-Sheng Ye, Wei Jiang

**Affiliations:** School of Electronic Information, Wuhan University, Wuhan 430072, China

**Keywords:** fine-grained, adversarial detection, perturbation classification, high-frequency component

## Abstract

Adversarial examples present a severe threat to deep neural networks’ application in safetycritical domains such as autonomous driving. Although there are numerous defensive solutions, they all have some flaws, such as the fact that they can only defend against adversarial attacks with a limited range of adversarial intensities. Therefore, there is a need for a detection method that can distinguish the adversarial intensity in a fine-grained manner so that subsequent tasks can perform different defense processing against perturbations of various intensities. Based on thefact that adversarial attack samples of different intensities are significantly different in the highfrequency region, this paper proposes a method to amplify the high-frequency component of the image and input it into the deep neural network based on the residual block structure. To our best knowledge, the proposed method is the first to classify adversarial intensities at a fine-grained level, thus providing an attack detection component for a general AI firewall. Experimental results show that our proposed method not only has advanced performance in AutoAttack detection by perturbation intensity classification, but also can effectively apply to detect examples of unseen adversarial attack methods.

## 1. Introduction

Deep learning technology has demonstrated excellent performance and has played a key role in many fields, such as finance, medical care, and public safety [1,2,3,4]. However, deep learning is also a double-edged sword, and its security issues have aroused widespread concern among researchers and engineers. Among them, the research on adversarial examples has gained momentum in recent years [5]. Combining a clean example with a slight adversarial perturbation yields an adversarial example. An adversarial attack is a term for this process of creating perturbations. Deep neural networks (DNNs) may produce incorrect predictions due to adversarial attacks, which may then cause decision-making or other subsequent sub-tasks to perform incorrectly. This phenomenon puts safety-sensitive tasks in a dangerous situation. As an illustration, attacking autonomous driving may cause misrecognition of traffic signs, failure to detect vehicles in front, and loss of seeing passing pedestrians [6,7,8]. It is easy to foresee that these misidentifications will significantly harm the safety of people and their property in many different industries. Therefore, research on adversarial attacks and defenses is in full swing to examine the causes and decrease the probability of misidentifications.

From an attack perspective, researchers are working on exposing the vulnerabilities of DNNs to explore their mechanisms and interpretability. There are many ways to classify attack methods, which can be divided into white-box, gray-box, and black-box attacks according to the degree of information acquisition of the attacked model or system. In a white-box attack, the adversary has complete knowledge of the DNN, including its design, weights, inputs, and outputs. Specifically, the generation of the adversarial instances involves resolving an optimization issue guided by the gradient of a model. Common white-box attacks include gradient-based attacks [9,10] and optimization-based attacks [11,12]. In a grey-box attack, the adversary has limited knowledge of the model, and it has access to the model’s training data, but knows nothing about the model architecture. Therefore, its goal is to replace the original model with an approximate model and then use its gradients to generate adversarial examples like in the white-box scenario. Finally, the adversary knows nothing about the model in a black-box attack. As a result, the adversary can produce adversarial examples by leveraging the input sample content and the transferability properties of the adversarial examples. Common black-box attacks include gradient estimation-based attacks [13] and decision boundary-based attacks [14].

The defense works are trying every means to solve exposed or potential loopholes to ensure the reliability and trustworthiness of DNNs in practical applications. There are numerous ways to categorize defense strategies, which can be separated into intrusive and non-intrusive methods depending on whether the protected model is modified. Intrusive methods mainly include modifying model regularization loss [15,16] and adversarial training [17,18]. The adversarial training or regularization method is computationally expensive, despite effectively solving the defensive problem. On whether to alter the prediction results, non-intrusive approaches could be primarily split into input transformation methods [19,20,21,22] and detection methods [23,24,25,26,27,28,29,30,31,32,33]. These techniques have a low computational cost and the ability to generalize across models, but their defense performance is often much weaker than that of intrusive methods.

**Figure 1:**
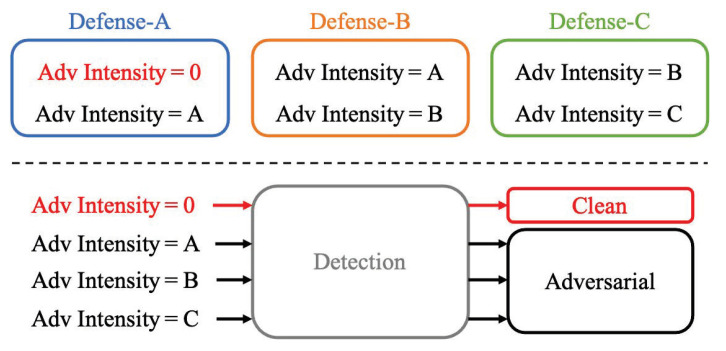
A diagram illustrating the characteristics of existing defense and detection methods.

Many adversarial defense strategies have already been developed, but as far as we can tell, they all have flaws. For instance, they may only be effective against adversarial examples with a specific range of perturbation intensities or may result in relatively high accuracy loss for clean inputs. In other words, even when defenses have been deployed, deep learning systems are still susceptible to attacks outside the range of an effective defense. The problem with limited defense range is shown at the top of Figure 1, where Defense-A is effective for adversarial intensities of 0 or A, and Defense-B and Defense-C can only defend against adversarial examples of some intensities. Meanwhile, existing adversarial detection methods only classify input samples into clean samples and adversarial examples [23,24,25,26,27,28,29,30,31,32,33], and there is no technique to classify the intensity of input samples, as shown at the bottom of Figure 1. To address the problem in Figure 1, a fine-grained adversarial detection (FAD) method with perturbation intensity classification is proposed in this paper. FAD is used to classify the potential perturbation intensities in the input samples to accurately provide adversarial examples with different perturbation intensities for different defense subtasks.

The main steps of the proposed FAD method are as follows: first of all, through the statistical observation of the spectrum of clean samples and adversarial examples with different intensities, this paper proposes an approach based on amplifying high-frequency components to improve the distinguishability of adversarial examples. The procedure is to use the Haar discrete wavelet transform (DWT) to decompose the high-frequency and low-frequency components, amplify the high-frequency components, and then use the inverse discrete wavelet transform (IDWT) to create the reconstructed image. Then, through the experimental observation of DNN to distinguish adversarial examples and refer to the existing methods of using DNN to detect adversarial examples, this paper proposes to use DNN to classify the perturbation intensity of the reconstructed image. This paper designs a 16-layer network based on the residual block structure [34] to achieve a fine-grained classification of adversarial intensity. This is done in light of the residual block structure’s excellent performance. Finally, the training sample adopts a representative AutoAttack method (AA) [35] to produce the training sample of the adversarial intensity classifier to obtain strong performance.

By studying the pattern of various intensity perturbations of AA in the feature space, the proposed FAD could extend to the fine-grained categorization of perturbation intensity of additional attack methods (especially high-frequency features). Our proposed method has a strong detection capability and can correctly classify the intensity of adversarial examples generated by different attack methods. The average classification accuracy of the proposed FAD technique, which detects different intensities of different adversarial attacks under the $l_{2}$ norm, is 98.95%.

The main contributions of this paper are as follows:

- To the best of our knowledge, FAD is the first fine-grained adversarial perturbation intensity classification method, and is a non-intrusive adversarial detection method.
- The FAD approach, which amplifies the high-frequency portion of the image, provides cross-model generalization capabilities as well as the ability to detect unseen and various mechanism adversarial attacks.
- We empirically demonstrate the feasibility of fine-grained perturbation intensity classification for adversarial examples, providing a detection component for general AI firewalls.

The rest of this paper is organized as follows. Section 2 presents the existing adversarial attack and defense methods. We describe our proposed method’s workflow and network architecture in Section 3. Section 4 describes the experimental details and compares the corresponding experimental results with other state-of-the-art (SOTA) detection methods. Furthermore, ablation studies are performed evaluating each component of the proposed defense. Finally, Section 5 concludes this work and provides an outlook on future fine-grained adversarial detection research.

## 2. Related Work

In this section, we review and summarize some existing methods for adversarial attacks and adversarial defenses. Furthermore, we briefly discuss the characteristics of these existing methods and the advantages of our proposed method.

### 2.1. Adversarial Attack

Numerous algorithms for adversarial attacks have been created by researchers. Goodfellow et al. proposed the fast gradient sign method (FGSM) [9], believing that the existence of adversarial examples is caused by the linear behavior of high-dimensional space. By executing a one-step calculation with step size epsilon in the gradient sign’s direction, FGSM realizes the generation of attack examples:

(1) $$x_{a}=x+\epsilon \cdot \mathrm{sign}\left(\nabla_{x}J\left(x,y\right)\right),$$

where *x* and $x_{a}$ represent clean samples and adversarial examples, respectively, *J* is the neural network’s loss function, and *y* is the corresponding real label. ϵ is a hyperparameter that controls the distance between clean samples and adversarial examples. Additionally, since FGSM is a single-step calculation attack method, the speed at which adversarial examples are generated is extremely fast. The FGSM attack works by producing an adversarial perturbation via increasing the gradient of the model, deceiving the classification model. However, FGSM often produces large perturbations and its attack success rate is insufficient.

On this basis, Madry et al. improved the step size and the number of iterations that increase in the opposite direction of the gradient sign, and proposed the projected gradient descent attack (PGD) [10]. Its general idea is to perform multiple iterations; each iteration has a smaller step size and projects the perturbation into its limited area. The specific implementation of PGD is as follows:

(2) $$x_{t+1}=\prod_{x+\epsilon }\left(x_{t}+\beta \cdot \mathrm{sign}\left(\nabla_{x}J\left(x_{t},y\right)\right)\right),$$

where $x_{t}$ represents a sample with an iteration number of *t*, and β is a hyperparameter that controls the step size of each iteration. ∏ is a projection function that controls the distance between the generated adversarial examples and the clean samples. Its function is to project the examples beyond the distance limit onto the l${}_{p}$ norm sphere with the clean sample *x* as the origin and the radius ϵ. To obtain effective adversarial examples, the PGD attack maximizes the difference between prediction results while minimizing the intensity of adversarial perturbations. In addition, PGD has a relatively slow calculation speed due to its iterative attack method.

Different from PGD, Carlini et al. proposed an optimization-based attack called a CW attack [11]. The CW attack introduces a new variable *w*, transforming the problem of optimizing the perturbation δ into optimizing *w* by defining:

(3) $$\delta =\frac{1}{2}\left(\tanh\left(w\right)+1\right)-x.$$

Because −1≤tanh(w)≤1, 0≤x+δ≤1 is established, so that the generated adversarial examples are constrained to not exceed the range of [0,1]. By introducing the above constraints into the loss function of PGD, the objective function of CW attack can be obtained. The perturbation generated by the CW attack is almost imperceptible to the human eye, and can adjust the confidence of the classification results. However, its calculation takes a longer time than general attacks, and it is less convenient to implement.

For the lack of diversity in attacks such as PGD, Croce et al. proposed two improved PGD methods and combined them with FAB [36] and SquareAttack [37] to form a strong complementary attack combination (AA) [35]. The APGD attacks proposed by Croce et al. adds a momentum update mechanism on the basis of PGD. Let $\theta^{\left(j\right)}$ be the step size of the *j*th iteration, $x^{\left(j\right)}$ be the example of the *j*th iteration, then the updated step size of APGD is:

(4) $$\begin{array}{cc} u^{\left(j+1\right)}= & \prod_{x+\epsilon }\left(x^{\left(j\right)}+\theta^{\left(j\right)}\nabla_{x}J\left(x^{\left(j\right)},y\right)\right)\\ x^{\left(j+1\right)}= & \prod_{x+\epsilon }\left(x^{\left(j\right)}+\gamma \cdot \left(u^{\left(j+1\right)}-x^{\left(j\right)}\right)\right.+\left(1-\gamma \right)\cdot \left(x^{\left(j\right)}-x^{\left(j-1\right)}\right), \end{array}$$

where $u^{\left(j+1\right)}$ is the example of the *j*th iteration generated by original PGD, and γ∈[0,1] (using γ=0.75) regulates the impact of the previous update on the current update. In addition, using cross-entropy loss and difference of logit ratio loss as attack variants, two improved attacks are constructed, namely APGD${}_{CE}$ and APGD${}_{DLR}$. Finally, the combined attack method AA is formed by combining these two APGD methods with the existing FAB and SquareAttack methods.

(5) $$\mathrm{AA}={\mathrm{APGD}}_{CE}+{\mathrm{APGD}}_{DLR}+\mathrm{FAB}+\mathrm{SquareAttack}.$$

With a reasonable computational expense, AA can produce positive attack results against models with a wide variety of architectures. AA is used as the benchmark for robustness evaluation because it is by far the most potent attack.

### 2.2. Adversarial Defense

#### 2.2.1. Intrusive Defense

Adversarial training and network modification are two common examples of intrusive defense. When Goodfellow et al. [9] proposed the FGSM attack, they also proposed for the first time an adversarial training method, that is, adding the adversarial examples generated by FGSM to the training set to achieve data enhancement. This method achieves a relatively effective defense, but the fly in the ointment is that this method needs to train DNN from scratch, which takes a long time. Wong et al. proposed FastAT [18], a fast one-step adversarial training method, which may lead to catastrophic overfitting, although it improves the speed of training from scratch. Lee et al. proposed a regularized defense method called GradDiv [15]. GradDiv uses the von Mises distribution to make the gradient distribution after adversarial training more sparse, which improves defense performance.

#### 2.2.2. Non-intrusive Defense

Non-intrusive defense is mainly divided into input transformation defense and adversarial detection. Among them, the input transformation defense preprocesses the input samples, and then inputs the processed image into the classifier to make it output correctly. Mustafa et al. [19] used super-resolution methods to add high-frequency components to images to eliminate perturbations in attack samples, and further suppressed noise through wavelet domain filtering. Although this method has cross-model defense capability, its overall defense performance is not good. Liu et al. [20] proposed a defense framework based on image compression to remove adversarial perturbations from input images through the process of JPEG compression and decompression. However, the defensive performance of this method on high-resolution datasets is unsatisfactory.

Adversarial detection methods divide the input samples into clean samples and adversarial examples, and reject the adversarial examples to input the classifier. Tian et al. proposed a sensitivity inconsistency detector (SID) [24], which uses the difference in sensitivity of different samples to the high curvature region of the decision boundary to distinguish adversarial examples from clean samples. Specifically, a dual model is trained in the weighted average wavelet transform domain. Next, determine the sensitivity characteristics of the adversarial example and the clean sample by calculating the difference between the predicted value of the dual model and the original model. Finally, a sensitive features-based adversarial example detector is trained. However, this detection technique performs poorly for tiny perturbations and is mostly effective for larger ones. According to Liu et al. [23], image features can be separated into explicit features that are easily understood by people and latent features that are incomprehensible to people but are used by DNNs. Then, they provide a feature filter (FF) [23] that uses a DCT transform domain approach to convert spatial domain picture pixels into frequency domain coefficients. Then, the FF retains the majority of the explicit coefficients while eliminating the high-frequency areas’ coefficients to remove the majority of the latent characteristics. Lastly, by contrasting the input image and its transformed image with the anticipated labels of the DNN. The input image is adversarial if the predicted labels do not match. The method performs well in detecting CW attacks, however, it does not perform satisfactorily for other types of adversarial examples. Harder et al. proposed a method called SpectralDef [25] that uses Fourier domain analysis of input images to distinguish clean samples from adversarial images. Specifically, the method applies a 2D discrete Fourier transform on each image and its adversarial version. For the obtained magnitude Fourier spectrum, a binary classifier is trained to detect adversarial attacks, where the classifier adopts a logistic regression model. SpectralDef performs better in the detection of larger perturbations, but performs poorly in the case of small ones.

Our proposed FAD is a non-intrusive method that has stronger cross-model generalization ability than intrusive methods. In contrast to input transformation defenses in non-intrusive methods, our method does not degrades the classification performance for clean samples. Moreover, compared with detection methods in non-intrusive methods, our method can not only distinguish whether an input sample is adversarial or not, but also fine-grained classify its intensity under the $l_{2}$ norm condition.

## 3. FAD Methods

This paper introduces powerful AA adversarial examples [35] in the training set of FAD, aiming to be able to detect the most powerful attacks. Training FAD with AA is anticipated to result in better generalization abilities, such as detecting unseen attacks, due to the diversity of AA components. The adversarial examples produced by AA are used in this section as the investigation object to conduct the following observations and hypotheses.

### 3.1. Observations and Hypotheses

#### 3.1.1. High-Frequency Component

**Figure 2:**
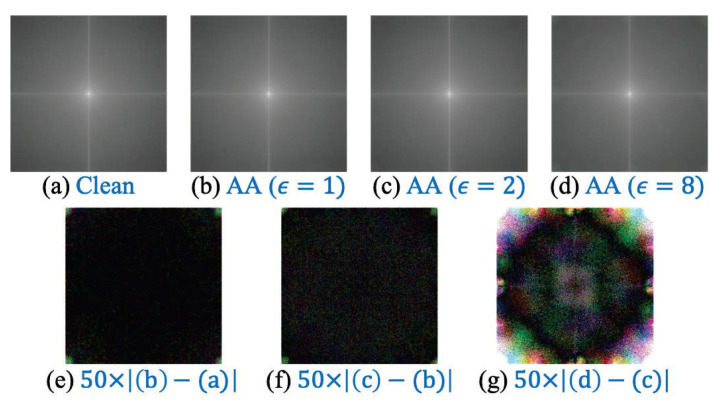
For the 10-category subset of the ImageNet dataset [42], use DFT to calculate the spectrum of the samples and average the results. (**a**) represents clean samples, (**b**) represents AA attack examples [35] with ϵ=1.0, and (**e**) indicates the result of magnifying the difference between (**b**,**a**) by 50 times.

Currently, there is no unified conclusion on the distribution characteristics of different adversarial examples. In the existing work, some studies posit that the adversarial perturbation is concentrated in the high-frequency region [21,22,38,39,40], and some suggest that its distribution is related to the dataset [41]. Is it possible to classify the intensity of adversarial examples via certain operations on high-frequency features? In this paper, we observe clean samples and adversarial examples from the frequency domain. Specifically, the frequency spectrum of all samples in the data set is calculated by discrete Fourier transform, then averaged and visualized. As shown in Figure 2a–d are the spectrum mean values of clean samples, the AA attack examples with ϵ=1, the AA attack examples with ϵ=2, and the AA attack examples with ϵ=8 (each 3000 samples). Then, (e) is the result of magnifying the difference between the spectrum of the AA attack sample with ϵ=1 and the clean sample by 50 times, (f) is the result of magnifying the difference of the spectrum of the AA attack sample with ϵ=2 and ϵ=1 by 50 times, and (g) is the result of magnifying the difference of the frequency spectrum of the AA attack samples with ϵ=8 and ϵ=2 by 50 times.

It is not difficult to find that all the spectral differences are more obvious in the high-frequency region (four corners), while the difference in the low-frequency region is slightly weaker. Especially in the spectrum difference between the AA attack samples with ϵ=8 and ϵ=2 (as shown in Figure 2a), the difference in the high-frequency region is particularly noticeable. Intuitively, extracting or enhancing high-frequency features will help distinguish adversarial examples of different intensities. In summary, after analyzing the characteristics of the ImageNet dataset [42] (a subset of 10 categories) and its adversarial versions with different intensities, it is found that the differences in high-frequency regions between samples with different adversarial intensities are obvious, while the differences in low-frequency regions are weak. Therefore, this paper believes that the characteristics of distinguishing adversarial examples are mainly related to high frequencies, and also include some low-frequency signals. Its essence is a high-level feature, and it happens to be the insensitive feature of the human vision system. Based on this, we hypothesize that by amplifying the high-frequency components of the image, the distinguishability of adversarial examples of varying intensities can be enhanced.

#### 3.1.2. Discrimination Ability of DNN

**Figure 3:**
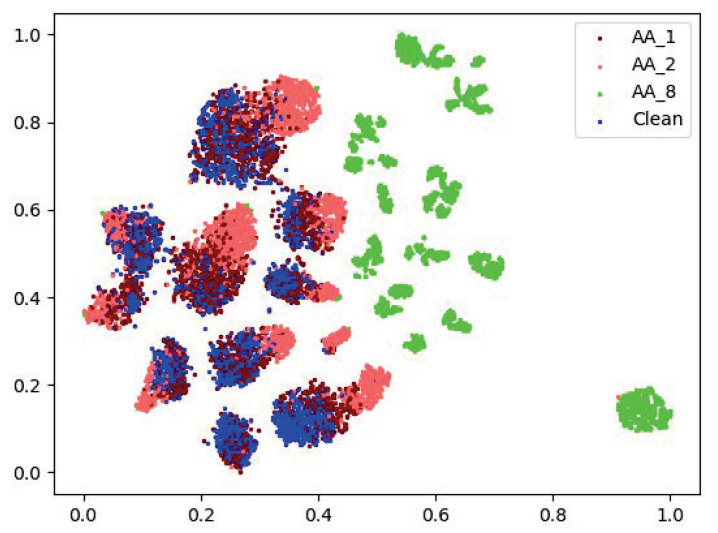
Input samples of different intensities into the base classifier ResNet-34 [34], and visualization of the output of the penultimate layer of ResNet-34 through T-SNE [43]. AA_1 means AA attack examples [35] with ϵ=1.0.

Some works utilize the features of the last few layers of DNN to train shallow detection networks [26,27,28,29,30,31,32], indicating that reasonable use of high-level features extracted by DNNs can distinguish adversarial examples from clean samples. Furthermore, [33] employs a DNN to directly perform end-to-end detection on samples. Since DNN can be used to detect adversarial examples, can DNN be used to classify the intensity of adversarial examples? In this paper, the AA attack is performed on the base classifier ResNet-34 (trained on the ImageNet-10 dataset), and the output (logits) of the penultimate layer is reduced by T-SNE [43] and visualized, as shown in Figure 3.

In this low-dimensional feature space, adversarial examples with ϵ=8 (green dots) are basically completely separated from other samples, and adversarial examples with ϵ=2 (red dots) can be separated from clean samples (blue dots) to a certain extent. As a result, this paper believes that adversarial examples of various intensities are more easily distinguishable in the feature space (non-reduced version) where DNN is located. Due to the ResNet-34’s task limitation (10-class classification) in Figure 3, it is unable to distinguish between adversarial examples of various intensities. On this basis, we hypothesize that the DNN’s capacity to discriminate can be utilized to specifically train a model for the discrimination of examples with various intensities.

### 3.2. Classifying the Adversarial Intensity

**Figure 4:**
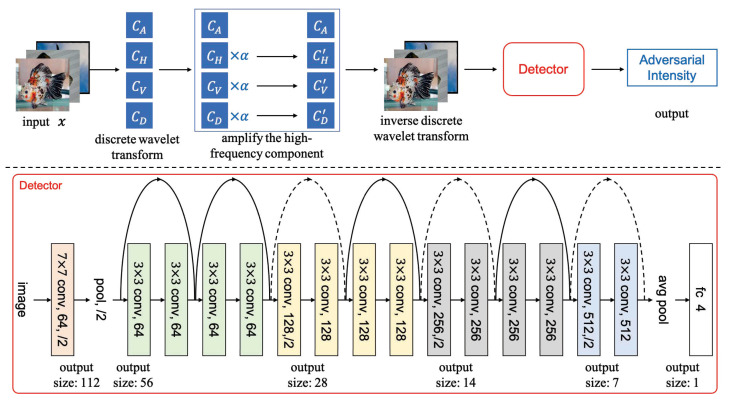
The top is the workflow diagram of the proposed FAD, and the bottom is the structural schematic of the proposed detector model.

While humans primarily employ low-frequency components to identify images, DNN can integrate high-frequency and low-frequency components of images for classification processing. This paper designs the FAD’s workflow (as shown in Figure 4) to detect and classify the intensity of adversarial examples by fusing the observations and hypotheses regarding high-frequency components and DNN discrimination ability in the previous subsection. Briefly, our proposed method first augments the high-frequency components of images, then trains a detector model using the enhanced images. Figure 4 depicts the detailed inference process. First, a single-level Haar DWT is applied to the input image to produce the approximation coefficient matrix ($C_{A}$), the horizontal detail coefficient matrix ($C_{H}$), the vertical detail coefficient matrix ($C_{V}$), and the diagonal direction coefficient matrix ($C_{D}$). The next step is to enhance the high-frequency components $C_{H}$, $C_{V}$, and $C_{D}$ to produce $C_{H}^{\prime }$, $C_{V}^{\prime }$, and $C_{D}^{\prime }$, respectively. The resulting $C_{A}$, $C_{H}^{\prime }$, $C_{V}^{\prime }$, and $C_{D}^{\prime }$ are then subjected to an IDWT to produce the enhanced image. Finally, the enhanced image is fed into the detector, which provides the predicted adversarial intensity.

#### 3.2.1. Enhance High-Frequency Components

For a 2-D image *X*, the 2-D DWT will perform a 1-D DWT on each row and column of *X*, that is

(6) $$\left\{\begin{array}{c} C_{A}=LXL^{T}\\ C_{H}=HXL^{T}\\ C_{V}=LXH^{T}\\ C_{D}=HXH^{T} \end{array}\right.,$$

where *H* and *L* represent the low-pass filter and high-pass filter of the orthogonal wavelet, respectively. In addition, $C_{A}$ is the low-frequency component of image *X*, which is an approximate image with lower resolution, including the basic object structure. $C_{H}$, $C_{V}$, $C_{D}$ are the high-frequency components of image *X*, which contain most of the image details including noise. The decomposition of the high-frequency and low-frequency components of the image is finished by the aforementioned operation, and the high-frequency components of the image will subsequently be enhanced.

(7) $$\left\{\begin{array}{c} C_{H}^{\prime }=\alpha \cdot C_{H}\\ C_{V}^{\prime }=\alpha \cdot C_{V}\\ C_{D}^{\prime }=\alpha \cdot C_{D} \end{array}\right.,$$

where α (α>1) is the enhancement coefficient of high-frequency components. Then apply IDWT to reconstruct the image, then the 2-d IDWT is defined as:

(8) $$X^{\prime }=L^{T}C_{A}L+H^{T}C_{H}^{\prime }L+L^{T}C_{V}^{\prime }H+H^{T}C_{D}^{\prime }H,$$

where $X^{\prime }$ represents the reconstructed image with enhanced high-frequency components. The single-level 2D-DWT in Python’s PyWavelets package [44] is used in this paper to compute the DWT and IDWT processes in (6) and (8).

#### 3.2.2. The Architecture of the Detector

**Figure 5:**
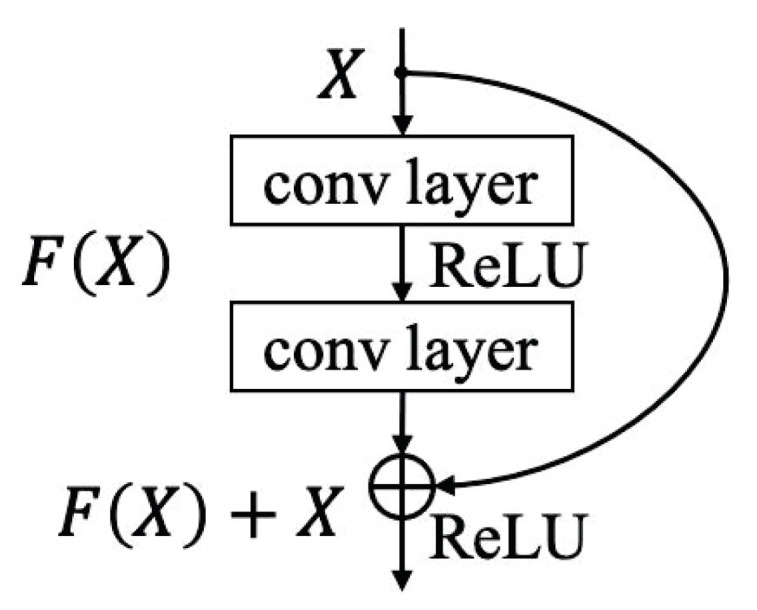
A schematic diagram of the structure of a residual block.

At the bottom of Figure 4, the detector model’s architecture is given. In order to accomplish fine-grained detection of adversarial intensities, a 16-layer network is constructed in this paper based on the residual block structure. This paper draws on the idea of ResNet and builds a residual block with “shortcut connection”. We also divide the network into 6 building layers based on this and in accordance with the ResNet model’s network architecture. A building layer can contain one or more network layers, and one or more residual blocks. The first building layer of the detector model is constructed by a normal convolution layer and a maximum pooling layer, and the second building layer consists of 2 residual blocks. The third, fourth, and fifth building layers all start with a downsampling residual module, followed by 2, 2, and 1 residual modules, respectively. Finally, the sixth building layer is constructed by a global pooling layer and a fully connected layer. The connection sequence of the various building layers is shown at the bottom of Figure 4. When the input and output dimensions are the same, a shortcut connection can be used directly (the solid line shortcut connection shown at the bottom of Figure 4). In order to match the new feature map size when the dimension grows (the dotted line shortcut connection shown at the bottom of Figure 4), a 1×1 convolution with a step size of 2 is employed. A residual block is shown in Figure 5.

Formally, a residual block is defined as:

(9) $$Y=F(X,W_{i})+X,$$

where the residual block’s input and output vectors are *X* and *Y* and the residual mapping to be learned is represented by the function $F(X,W_{i})$, and $W_{i}$ is the weight of the *i*th convolution layer. For instance, there are two layers of convolution in Figure 5, which can be expressed as $F=W_{2}\sigma \left(W_{1}X\right)$, σ represents the ReLU function, bias is ignored for simplicity, and the operation of F+X is completed by shortcut connection. After the addition is finished, a second non-linear ReLU function is applied. However, the dimensions of *F* and *X* in (9) must be the same, and if not, an additional matrix $W_{d}$ (using a 1 × 1 convolution with a stride of 2) can be multiplied with *X* to match the dimensions of *F*:

(10) $$Y=F\left(X,W_{i}\right)+W_{d}X.$$

In order to achieve a faster calculation speed, the residual block designed in this paper only involves the function *F* of 2-layer convolution.

The 16-layer network we created is referred described as a “fine-grained detection network” (or simply “detector”). In order to train the detector, we first train a base classifier model on a regular (non-adversarial) dataset as usual, and then use the AA attack against the base classifier to generate adversarial examples of different intensities for each data point of the training set. A balanced multivariate classification dataset that is n+1 times larger than the initial dataset is then obtained (*n* is the number of intensities of the set adversarial perturbations). This dataset consists of raw data (label 0) and corresponding adversarial examples (label *k*, k∈[1,n]). Finally, the detector is trained to reduce the cross-entropy between input samples and labels.

## 4. Experiments

### 4.1. Experiment Settings

#### 4.1.1. Adversarial Attack Methods

To evaluate the effectiveness of the proposed FAD, we adopt four classic adversarial attack methods (under $l_{2}$-norm) mentioned in Section 2: AA [35], FGSM [9], PGD [10], and CW [11]. Furthermore, to achieve different levels of attack effects, we divide FGSM, PGD, and AA attacks into three different intensities according to the attack success rate, and CW is set to one intensity with a high attack success rate. As for the ImageNet dataset, we set the perturbation intensities ϵ of the FGSM, PGD, and AA attacks to 1.0, 2.0, and 8.0, respectively. Furthermore, we limit the intensity of the CW attack to ϵ=2.0 because of its long computation time. The max iterations of the CW attack are set to 20, and the binary search steps are set to 3. FGSM, PGD, and CW are implemented by a PyTorch library CleverHans [45]. We use the standard version of AA through the official open-source code.

#### 4.1.2. Datasets

This work restricts the non-adversarial classification dataset to ten categories in order to keep the computational resources necessary to generate modest adversarial examples and to prevent having similar categories which are too similar, which would oversimplify the attack’s task. To test the effectiveness of the proposed method, we conduct experiments on a subset of the ImageNet dataset [42], called ImageNet-10, that contains all the data from ten classes that were chosen randomly. We picked 10,000 clean images (10 categories in total, 1000 images in each category) from the ImageNet-10 training set. For each category, we randomly selected 600 images as the training set, 200 as the validation set, and 200 as the test set to train the target model. All images from the ImageNet-10 are resized to 224×224×3. The base classifier chosen for this work is ResNet34 [34], trained on ImageNet-10. Of the above 10,000 images, 3224 were successfully attacked by AA (against ResNet-34) with ϵ=1.0,2.0, and 8.0, and these images were used to train our detector. In addition, we selected 3000 clean samples (300 for each category) from the verification set, and obtained 410 successfully attacked images after using 3 intensities of AA attacks to evaluate our detector.

#### 4.1.3. Training

All training processes are performed on an Nvidia GeForce RTX 2080Ti GPU with an Intel Core i9-10900X CPU. For all models, the number of total training epochs is 100. The batch size is set to 8 for all detector models when training on the adversarial dataset. In addition, the batch size is set to 32 for base classifier when training on the ImageNet-10 dataset. Adam is used as the base classifier’s optimizer while SGD is used as the detector’s optimizer. Meanwhile, the learning rates of all base model and detector models are set to $3\times 10^{-4}$. FAD’s high-frequency component enhancement coefficient α is set to 2. We trained each model involved in the experiments five times under each parameter setting, and selected the best one to complete the evaluations.

### 4.2. Experimental Result

#### 4.2.1. Fine-Grained Perturbation Intensity Classification

Table 1 shows our proposed method’s fine-grained classification performance for various intensities of adversarial examples. The items in column 1 represent different input samples (test sets for evaluation); for example, “AA${}_{\epsilon =1}$” means AA adversarial examples with ϵ=1.0. Among them, all adversarial examples are generated by attacking the ResNet-34 trained on the ImageNet-10 dataset. In Table 1, the first row represents the output of FAD. “Level-0” means FAD’s predicted value is a clean sample (whose adversarial intensity is ϵ=0), while “Level-1”, “Level-2”, and “Level-8” represent predicted adversarial intensities are ϵ=1.0, 2.0, and 8.0, respectively. For example, the value of “AA${}_{\epsilon =1}$” with “Level-2” indicates the probability that FAD predicts AA examples with ϵ=1.0 as samples with an adversarial intensity of ϵ=2.0. The training set of FAD only includes clean samples and AA attack examples. It can be seen that the fine-grained classification performance of our proposed FAD on the test set is excellent, especially for clean samples and AA examples with ϵ=8.0, both can achieve 100% accuracy. The classification accuracy for the AA examples with ϵ=1.0 can also reach 99.76%, and there is only a 0.24% chance that they will be incorrectly identified as clean samples. In addition, for the AA examples with ϵ=2.0, the classification accuracy is 98.78%, and there is only a 1.22% probability of misclassifying them as “Level-1” attack examples. Furthermore, the classification accuracy; the FGSM and PGD examples (ϵ=1.0, 2.0, and 8.0) are all greater than 96%.

FAD exhibited a high level of classification accuracy for the FGSM and PGD examples that were not included in the training, proving that it is capable of classifying the intensities of unseen attack examples and that its performance is potent.

#### 4.2.2. Comparing with Other Detection Methods

**Figure 6:**
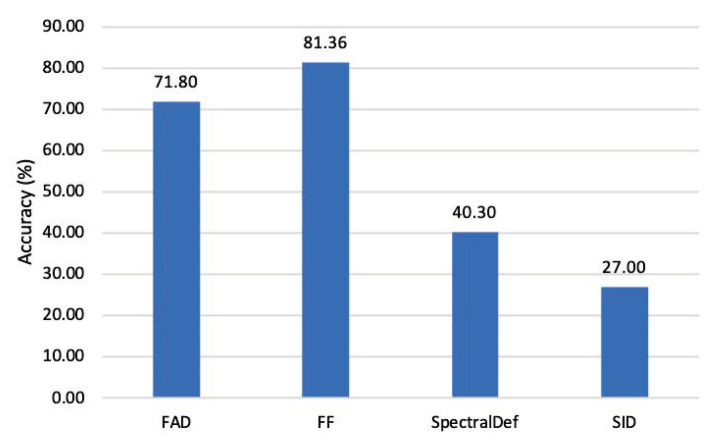
Detection accuracy (%) of FAD and the SOTA methods for CW attack examples [11] generated by attacking ResNet-34.

In this paper, in the detection of different attack examples, the current SOTA methods (SID [24] and SpectralDef [25]) are selected for comparison to evaluate the performance of our proposed FAD. Additionally, this work includes FF [23] for comparison only when detecting CW attack examples, since the FF method is specifically designed for the task. This work investigates the detection performance of FAD and the SOTA detection method in Table 2 and Figure 6, since the existing SOTA detection methods mainly detect whether the input samples are adversarial.

As seen in Table 2, SpectralDef’s accuracy for the detection of FGSM with ϵ=8.0 is 96.10%, which is significantly better than SID’s accuracy of 67.80%. The SpectralDef method has the advantage that it performs better at detecting adversarial examples with ϵ=8.0 and has accuracy larger than 96%, but it performs worse at detecting clean samples and adversarial examples with low intensity. In addition, SID has a good detection effect on clean samples, as well as AA and PGD examples with ϵ=2.0 and 8.0, all greater than 83%. Especially for AA and PGD examples with ϵ=8.0, the detection accuracy can reach 100%, but the detection performance is poor for FGSM examples, AA examples with ϵ=1.0, and PGD examples with ϵ=1.0. It is easy to realize that FAD outperforms SpectralDef and SID at detecting various samples. Moreover, SID performs better than SpectralDef in all but one situation: the detection of FGSM examples with ϵ=8.0. It is worth mentioning that all detection methods are trained using a dataset of clean samples and AA attack examples, and they can be extended to detect previously unseen attack examples to some extent.

Figure 6 compares the effectiveness of FAD, FF, SpectralDef, and SID in detecting CW attack examples, where the CW attack examples are produced by attacking ResNet-34. The accuracy of FAD, which is second only to the FF’s accuracy of 81.36% in this group, is 71.80% when it comes to detecting CW examples. SpectralDef and SID perform slightly worse in terms of detecting CW examples, with accuracy of less than 50%. Furthermore, only FF does not need training, and none of the other methods have ever used CW examples during training. As a result, FAD, SpectralDef, and SID may all be extended to detect CW examples to some extent, with FAD having a stronger generalization ability.

To sum up, we believe that the significant detection effect of FAD on unseen attacks (PGD, FGSM, and CW) may be because it has learned the common features of adversarial examples in high-frequency regions. Further, some mechanism similarities between AA, PGD, and FGSM may result in a similar distribution of the final optimized adversarial examples. The similarity between CW and AA is small, which may account for the slightly diminished effectiveness of detecting unseen CW adversarial examples.

#### 4.2.3. Cross-Model Detection

This work generates adversarial examples of various intensities against GoogLeNet [46] (trained on ImageNet-10) and utilizes these examples to evaluate FAD to investigate if it can successfully detect adversarial examples produced by attacking unseen models, as shown in Table 3. Here, FAD, SpectralDef, and SID are all trained with clean samples and AA attack examples generated by attacking ResNet-34. It is clear that SID essentially lacks cross-model identification capabilities and only performs well for clean samples. In contrast to SID, FAD and SpectralDef have some degree of cross-model detection capability. Furthermore, SpectralDef has a higher detection accuracy for attack samples with low intensities (ϵ=1.0 and 2.0), all of which are above 34%. For clean samples and attack instances with ϵ=8.0, FAD has stronger detection performance, and its accuracy is larger than 95%, particularly for PGD examples with ϵ=8.0, where the accuracy can exceed 100%. However, FAD’s detection ability is subpar for low-intensity attack examples, with accuracy not exceeding 3%.

In brief, FAD can detect high-intensity adversarial examples generated by attacking the unseen model. We believe that this phenomenon is caused by the similar boundaries of different models of the same task [47], so the distribution of adversarial examples is relatively similar.

#### 4.2.4. Applying to the L${}_{\infty }$ Norm

This work generates $l_{\infty }$ norm adversarial examples of various intensities against ResNet-34 and utilizes these examples to evaluate FAD to investigate if it can successfully detect adversarial examples under different norm constraints, as shown in Table 4.

Among them, FAD, SpectralDef, and SID all use clean samples and $l_{2}$ norm AA examples generated by attacking ResNet-34 for training. Observing the “L${}_{2}$ Norm” column, the average perturbation intensities of the $l_{\infty }$ norm attack examples in Table 4, after converting to the $l_{2}$ norm, are all greater than 32.40. At this time, SID only has a better detection performance for PGD examples with ϵ=8/255 under the $l_{\infty }$ norm, and its accuracy rate is 91.47%. In the remaining cases, the detection accuracy of SID was less than 50%. In contrast, SpectralDef has a detection accuracy higher than 96% for adversarial examples of various strengths under $l_{\infty }$ norm. For the FGSM and PGD examples with ϵ=8/255 under $l_{\infty }$ norm, SpectralDef’s detection accuracy can reach 100%. Moreover, FAD applied to $l_{\infty }$ norm has better performance in adversarial example detection, and its detection accuracy for attack examples with different intensities was higher than 99%.

In general, FAD is better than SpectralDef and SID at detecting unseen $l_{\infty }$ norm attack examples of varying intensities. It is noteworthy that all methods above can extend to the detection of $l_{\infty }$ norm attack examples to some extent.

#### 4.2.5. Applying to Image Segmentation Task

Existing work not only evaluates the adversarial robustness of image classification tasks but also focuses on other image analysis tasks, such as image segmentation and landmark detection [48]. Therefore, this paper evaluates whether the proposed FAD can be extended to image segmentation tasks, as shown in Table 5. In detail, the image segmentation model uses a pre-trained FCN_ResNet50 [49] obtained from the TorchVision library. The FCN_ResNet50 is trained on a subset of COCO [50] using only 20 classes from the Pascal VOC dataset [51]. To reduce computational costs, a subset of ImageNet-10, using only four categories (“airplane”, “bird”, “car”, and “dog”) present in the Pascal VOC dataset, is selected to implement training and evaluation in this case. The training set (called Segment-Trainset) contains 1187 clean samples, and the FGSM examples with ϵ=1.0, 2.0 and 8.0 (1187 attack examples per intensity) generated by attacking FCN_ResNet50. The test set, called Segment-Testset, contains 139 clean samples, and the FGSM examples with ϵ=1.0, 2.0, and 8.0 (139 attack examples per intensity) are generated by attacking FCN_ResNet50, in which “FAD_Cls” denotes the FAD trained on the image classification dataset (ImageNet-10), and “FAD_Seg” denotes the FAD trained on the image segmentation dataset (Segment-Trainset).Both “FAD_Cls” and “FAD_Seg” are evaluated on the Segment-Testset dataset.

As can be seen from Table 5, “FAD_Cls” can hardly detect the attack samples against the image segmentation model, and its detection accuracy is only 0.72%. Compared with “FAD_Cls”, “FAD_Seg” can effectively detect the attack samples against the image segmentation model. In particular, the detection accuracy of “FAD_Seg” reaches 99.28% for the FGSM samples with ϵ=2.0 and 8.0. In general, the adversarial sample detection for the image segmentation task cannot be performed using the FAD trained on the image classification dataset (“FAD_Cls”). However, the FAD trained from scratch on the image segmentation dataset (“FAD_Seg”) can be effectively applied to the adversarial sample detection for the image segmentation task.

#### 4.2.6. White-Box Attack against FAD

Up to this point, the performance evaluation of FAD in this paper has been based on the premise that the detector will not be attacked. If the attacker has complete knowledge about the detector, then the detector is vulnerable to attack and may output wrong results [52]. Therefore, we design the experiments of white-box attacks against FAD in Table 6. A PGD attack is used to evaluate the performance of FAD when FAD is completely transparent to the attacker. “Non-Targeted Attack” indicates that the class of expected output after being attacked is not specified, and “Targeted Attack” is the opposite.

From Table 6, the effectiveness of the targeted attack far exceeds that of the non-targeted attack, which makes the FAD detection accuracy 0% at ϵ=1, 2, and 8. In the case of non-targeted attacks, the detection accuracy of FAD decreases rapidly as the attack intensity gradually increases. The results show that if the attacker has complete knowledge about the FAD, then the output of the FAD will be left to the manipulation of the attacker (in the case of the targeted attack). In practical applications, the attacker usually knows only part of the detector’s information, and the actual performance of the FAD against the attacker will be better than in the case of Table 6.

### 4.3. Ablation Study

#### 4.3.1. The Value of α

**Figure 7:**
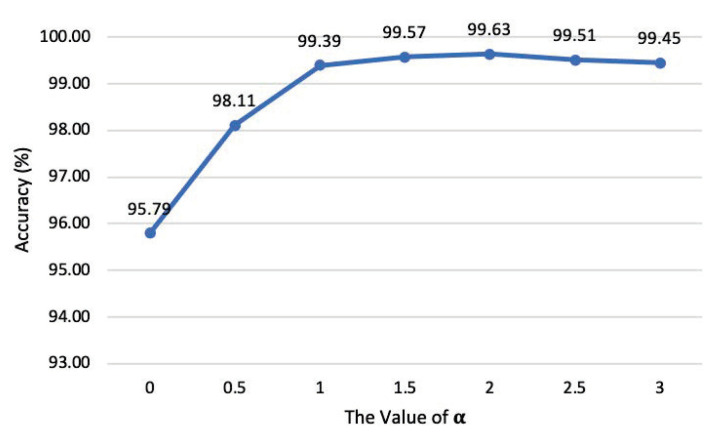
The relationship between the classification accuracy of FAD and the value of high-frequency enhancement coefficient α.

We explored the classification effect of FAD for clean samples and different intensities of AA examples under different α values through experiments, as shown in Figure 7.

Among them, α=0,0.5,1,1.5,2,2.5, and 3, while α=1 means that no processing is performed on the sample. As mentioned above, when α>1, it is to gain the input sample‘s high-frequency component, to select an appropriate high-frequency component gain coefficient to improve the classification performance of adversarial example intensities. It can be seen from Figure 7 that the performance is the best when α=2, which can make the average classification accuracy of attack examples with different intensities reach 99.63%, which is slightly higher than the case of not doing any processing on the input sample (α=1). To investigate the importance of the high-frequency component in the classifying of the intensities of the adversarial example, the high-frequency component of the sample is attenuated by setting α<1. Obviously, adversarial example intensity classification suffers greatly from the attenuation of high-frequency components. When α=0, the average classification accuracy for adversarial intensities drops to 95.79%.

#### 4.3.2. Selection of Transformations

**Figure 8:**
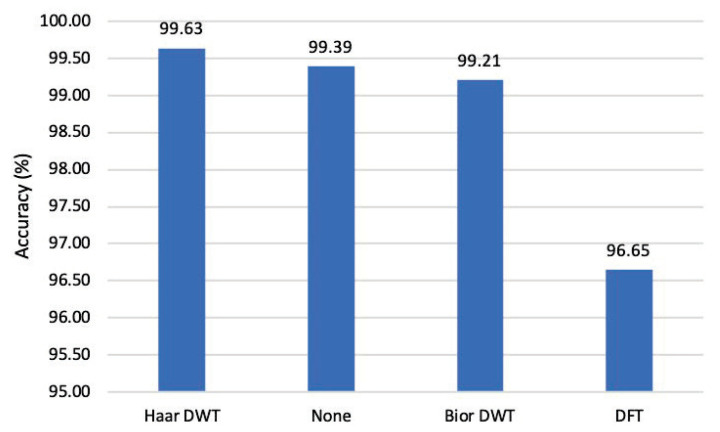
FAD’s classification accuracy when different transformations are used to separate the image’s high-frequency and low-frequency components.

This paper designs an experiment to investigate the classification accuracy of FAD for AA examples of different intensities and clean samples when using different transformation methods to separate high-frequency and low-frequency components, as shown in Figure 8.

Among them, using Haar DWT to decompose high-frequency and low-frequency components has the highest classification accuracy rate of 99.63%. Furthermore, the classification accuracy using Bior1.3 DWT is 99.21%, lower than 99.39% without any transformation (the bar corresponding to “None” in Figure 8). However, the performance degradation of the method using discrete Fourier transform is obvious, and the accuracy rate is 96.65%.

Generally, the Haar wavelet transform method adopted by FAD has a more significant contribution than other transforms to classify adversarial examples with different intensities.

#### 4.3.3. Selection of Training Examples

We verified the classification effect of FAD on various seen and unseen adversarial examples with different intensities when examples generated by varying attack methods were used in the training set, as shown in Table 7. By adding different intensities of Gaussian noise to clean samples, the samples generated to train FAD have better performance in classifying clean samples, AA examples with ϵ=8.0, and PGD examples with ϵ=8.0, and poor performance in the rest of the cases. The best result is achieved when using AA attack examples as training data for the classifying of clean samples and AA examples of varying intensities. At the same time, for the classification of FGSM examples of different intensities, the best performance is the case of using FGSM attack examples for training. Unsurprisingly, training with PGD examples yields the greatest results for classifying PGD examples of various intensities.

In conclusion, it can be generalized to identify examples produced by other unseen attack methods to some extent by using AA, FGSM, or PGD attack examples as training samples. Furthermore, our proposed FAD uses AA attack examples as training samples, and its average classification accuracy (98.95%) is higher than that of using other attack examples (or Gaussian noise samples).

#### 4.3.4. Detectors Using Various Network Architectures

**Figure 9:**
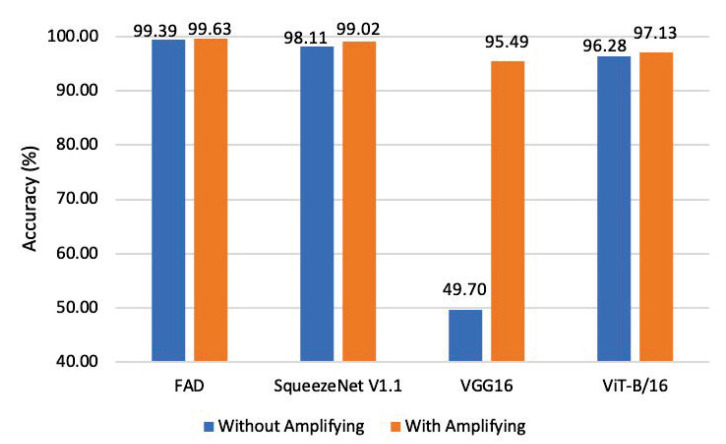
Classification accuracy on adversarial example intensity when detectors using various architectures of DNNs.

This paper designs experiments to compare the adversarial intensities (clean samples and AA examples) classification effect of our proposed 16-layer network (corresponding to the “FAD” in Figure 9) and other different architecture models (SqueezeNet V1.1 [53], VGG16 [54], and ViT-B/16 [55]) as the detector model, as shown in Figure 9.

At the same time, the experiments evaluate how well each detector model performs when the high-frequency component is enhanced or not.

All networks’ classification accuracy has increased after enhancing the high-frequency component, but VGG16’s accuracy has grown the most, from 49.70% to 95.49%. In addition, the accuracy of the proposed FAD is higher than that of the other three networks with different architectures as detectors, which verifies that our proposed 16-layer network has a clear advantage in the classification task of different intensity adversarial examples.

#### 4.3.5. Detectors with Different Layer Numbers

**Figure 10:**
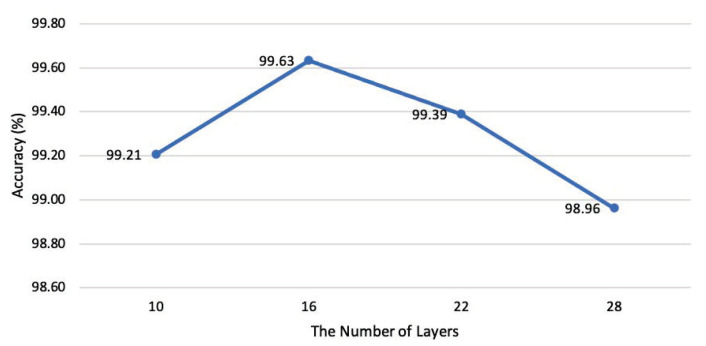
Classification accuracy of FAD when using residual block-based networks with different number of layers as detectors.

We design experiments to compare the classification effect of FAD on different intensity AA examples and clean samples when using different layer numbers of residual block-based networks (proposed in Section 3) as the detector model, as shown in Figure 10.

At the same time, the specific structure of the network with different layer numbers involved in the evaluation has shown in Table 8. Among the four networks with different numbers of layers, the 16-layer network achieved the highest average classification accuracy (99.63%), while the 28-layer network achieved the lowest average classification accuracy (98.96%).

Therefore, in the network design based on the residual block structure, the 16-layer network is most suitable for the classification task of adversarial examples with different strengths. In other words, the deeper the network does not mean the better the performance of the above tasks. The neural architecture search method might create an effective network for the classification task, but the cost is too high and will not be covered here.

## 5. Conclusions and Discussion

In this work, we efficiently classify adversarial examples of various intensities by augmenting the high-frequency components of the image and feeding the augmented image into our constructed DNN based on the residual block structure. Moreover, the proposed FAD performs well for classifying $l_{2}$ norm attack examples with varying intensities in a fine-grained manner and can be applied as a detection component of a general AI firewall. Compared to the SOTA adversarial detection method, FAD has superior detection performance and could even be used to detect $l_{\infty }$ norm adversarial examples. Furthermore, FAD is extensible, as it can be applied to image classification tasks as well as to other image analysis tasks (e.g., image segmentation) for adversarial sample detection.

At the same time, the FAD method can detect the adversarial examples generated by the unseen attack method and can be generalized to the adversarial examples generated by attacking the unseen model. Existing attack methods for identifying traffic sign classifiers can cause the classifier to incorrectly predict a “Speed Limit 30” sign as a “Speed Limit 80” sign by adding shadows. In this classification task case, the use of our method can potentially reduce the number of rejections of adversarial samples (compared to the current detection methods). In addition, combining our method with defense methods applicable to different intensities has the potential to increase the proportion of correctly identified frames among several consecutive frames, improving the stability and reliability of DNN continuous decision-making. For future studies, we intend to investigate a more accurate distinguishing of different intensities of adversarial examples in the transform domain to obtain a more general fine-grained categorization of adversarial example intensities.

## Figures and Tables

**Table 1:** Fine-grained classification probability (%) of FAD for adversarial examples generated by attacking ResNet-34.

	Level-0	Level-1	Level-2	Level-8
**Clean**	**100.00**	0.00	0.00	0.00
**AA${}_{\epsilon =1}$** [35]	0.24	**99.76**	0.00	0.00
**AA${}_{\epsilon =2}$** [35]	0.00	1.22	**98.78**	0.00
**AA${}_{\epsilon =8}$** [35]	0.00	0.00	0.00	**100.00**
**FGSM${}_{\epsilon =1}$** [9]	1.71	**98.29**	0.00	0.00
**FGSM${}_{\epsilon =2}$** [9]	0.00	2.68	**97.32**	0.00
**FGSM${}_{\epsilon =8}$** [9]	0.00	0.00	0.00	**100.00**
**PGD${}_{\epsilon =1}$** [10]	0.98	**99.02**	0.00	0.00
**PGD${}_{\epsilon =2}$** [10]	0.00	3.66	**96.34**	0.00
**PGD${}_{\epsilon =8}$** [10]	0.00	0.00	0.00	**100.00**

**Table 2:** Detection accuracy (%) of FAD and the SOTA methods for adversarial examples generated by attacking ResNet-34. All methods are trained and evaluated with the $l_{2}$ norm attack examples.

	FAD	SpectralDef [25]	SID [24]
**Clean**	**100.00**	63.66	92.20
**AA${}_{\epsilon =1}$** [35]	**99.76**	40.49	66.83
**AA${}_{\epsilon =2}$** [35]	**100.00**	50.00	86.83
**AA${}_{\epsilon =8}$** [35]	**100.00**	97.32	**100.00**
**FGSM${}_{\epsilon =1}$** [9]	**98.29**	40.00	44.88
**FGSM${}_{\epsilon =2}$** [9]	**100.00**	47.80	57.56
**FGSM${}_{\epsilon =8}$** [9]	**100.00**	96.10	67.80
**PGD${}_{\epsilon =1}$** [10]	**99.02**	40.49	62.93
**PGD${}_{\epsilon =2}$** [10]	**100.00**	50.49	83.66
**PGD${}_{\epsilon =8}$** [10]	**100.00**	97.56	**100.00**

**Table 3:** Detection accuracy (%) of FAD and the SOTA methods for adversarial examples generated by attacking GoogLeNet [46]. All methods are trained and evaluated with the $l_{2}$ norm attack examples.

	FAD	SpectralDef [25]	SID [24]
**Clean**	**99.63**	66.09	93.12
**AA${}_{\epsilon =1}$** [35]	0.49	**35.14**	6.51
**AA${}_{\epsilon =2}$** [35]	2.33	**37.22**	7.00
**AA${}_{\epsilon =8}$** [35]	**99.75**	74.69	15.23
**FGSM${}_{\epsilon =1}$** [9]	0.37	**34.89**	6.39
**FGSM${}_{\epsilon =2}$** [9]	0.74	**36.36**	5.77
**FGSM${}_{\epsilon =8}$** [9]	**95.58**	68.43	10.81
**PGD${}_{\epsilon =1}$** [10]	0.49	**35.38**	6.51
**PGD${}_{\epsilon =2}$** [10]	2.58	**37.96**	6.63
**PGD${}_{\epsilon =8}$** [10]	**100.00**	80.71	16.34

**Table 4:** Detection accuracy of FAD and the SOTA methods for $l_{\infty }$ norm attack examples generated by attacking ResNet-34.The column “L${}_{2}$ Norm” indicates the value of converting the perturbation intensity of the $l_{\infty }$ examples into the $l_{2}$ norm. All methods are trained with the $l_{2}$ norm attack examples and evaluated with the $l_{\infty }$ norm attack examples.

Data	L${}_{2}$ Norm	FAD	SpectralDef [25]	SID [24]
**FGSM${}_{\epsilon =1/255}$** [9]	34.66	**99.20%**	96.87%	13.57%
**FGSM${}_{\epsilon =2/255}$** [9]	39.22	**100.00%**	98.63%	24.93%
**FGSM${}_{\epsilon =8/255}$** [9]	59.77	**100.00%**	**100.00%**	44.23%
**PGD${}_{\epsilon =1/255}$** [10]	32.40	**99.57%**	96.87%	19.13%
**PGD${}_{\epsilon =2/255}$** [10]	36.90	**100.00%**	98.70%	46.90%
**PGD${}_{\epsilon =8/255}$** [10]	51.89	**100.00%**	**100.00%**	91.47%

**Table 5:** For the image segmentation task detecting accuracy (%) of FAD trained on the image classification dataset (“FAD_Cls”) and FAD trained on the image segmentation dataset (“FAD_Seg”). All methods are trained and evaluated with the $l_{2}$ norm attack examples.

	FAD_Cls	FAD_Seg
**Clean**	**100.00**	94.24
**FGSM${}_{\epsilon =1}$** [9]	0.72	**95.68**
**FGSM${}_{\epsilon =2}$** [9]	0.72	**99.28**
**FGSM${}_{\epsilon =8}$** [9]	0.72	**99.28**

**Table 6:** Detection accuracy (%) of FAD after being attacked by non-targeted and targeted attacks. All methods are trained and evaluated with the $l_{2}$ norm attack examples.

	Non-Targeted Attack	Targeted Attack
**PGD${}_{\epsilon =1}$** [10]	**100.00**	0.00
**PGD${}_{\epsilon =2}$** [10]	**3.90**	0.00
**PGD${}_{\epsilon =8}$** [10]	0.00	0.00

**Table 7:** The classification accuracy (%) of FAD when examples created by different attack methods are used to train FAD. All methods are trained and evaluated with the $l_{2}$ norm attack examples.

	AA [35]	FGSM [9]	PGD [10]	Gauss
**Clean**	**100.00**	99.02	99.51	99.76
**AA${}_{\epsilon =1}$** [35]	**99.76**	97.07	98.78	5.37
**AA${}_{\epsilon =2}$** [35]	**98.78**	98.27	**98.78**	0.73
**AA${}_{\epsilon =8}$** [35]	**100.00**	**100.00**	**100.00**	76.59
**FGSM${}_{\epsilon =1}$** [9]	98.29	**98.78**	98.54	7.07
**FGSM${}_{\epsilon =2}$** [9]	97.32	**99.51**	91.46	0.98
**FGSM${}_{\epsilon =8}$** [9]	**100.00**	**100.00**	92.93	13.66
**PGD${}_{\epsilon =1}$** [10]	**99.02**	96.10	**99.02**	8.05
**PGD${}_{\epsilon =2}$** [10]	96.34	95.29	**98.05**	0.98
**PGD${}_{\epsilon =8}$** [10]	**100.00**	**100.00**	**100.00**	91.95
**Average**	**98.95**	98.40	97.71	30.51

**Table 8:** Residual block-based networks with different number of layers.

Layer Name	Output Size	10-Layer	16-Layer	22-Layer	28-Layer
conv1	112 × 112	3 × 3, 64, stride 2			
		3 × 3 max pool, stride 2			
conv2_x	56×56	$\left[\begin{array}{c} 3\times 3,64\\ 3\times 3,64 \end{array}\right]\times 1$	$\left[\begin{array}{c} 3\times 3,64\\ 3\times 3,64 \end{array}\right]\times 2$	$\left[\begin{array}{c} 3\times 3,64\\ 3\times 3,64 \end{array}\right]\times 3$	$\left[\begin{array}{c} 3\times 3,64\\ 3\times 3,64 \end{array}\right]\times 4$
conv3_x	28×28	$\left[\begin{array}{c} 3\times 3,128\\ 3\times 3,128 \end{array}\right]\times 1$	$\left[\begin{array}{c} 3\times 3,128\\ 3\times 3,128 \end{array}\right]\times 2$	$\left[\begin{array}{c} 3\times 3,128\\ 3\times 3,128 \end{array}\right]\times 3$	$\left[\begin{array}{c} 3\times 3,128\\ 3\times 3,128 \end{array}\right]\times 3$
conv4_x	14×14	$\left[\begin{array}{c} 3\times 3,256\\ 3\times 3,256 \end{array}\right]\times 1$	$\left[\begin{array}{c} 3\times 3,256\\ 3\times 3,256 \end{array}\right]\times 2$	$\left[\begin{array}{c} 3\times 3,256\\ 3\times 3,256 \end{array}\right]\times 2$	$\left[\begin{array}{c} 3\times 3,256\\ 3\times 3,256 \end{array}\right]\times 3$
conv5_x	7×7	$\left[\begin{array}{c} 3\times 3,512\\ 3\times 3,512 \end{array}\right]\times 1$	$\left[\begin{array}{c} 3\times 3,512\\ 3\times 3,512 \end{array}\right]\times 1$	$\left[\begin{array}{c} 3\times 3,512\\ 3\times 3,512 \end{array}\right]\times 2$	$\left[\begin{array}{c} 3\times 3,512\\ 3\times 3,512 \end{array}\right]\times 3$
	1×1	average pool, 4-d fc, softmax			

## Data Availability

Not applicable.

## Abbreviations

The following abbreviations are used in this manuscript:

DNNs	Deep neural networks
DWT	Discrete wavelet transform
IDWT	Inverse discrete wavelet transform
AA	AutoAttack method
SOTA	State-of-the-art
FGSM	Fast gradient sign method
PGD	Projected gradient descent attack
SID	Sensitivity inconsistency detector
FF	Feature filter method
